# SpSIZ1 from hyperaccumulator *Sedum plumbizincicola* orchestrates SpABI5 to fine-tune cadmium tolerance

**DOI:** 10.3389/fpls.2024.1382121

**Published:** 2024-07-08

**Authors:** Yuhong Li, Zhengquan He, Jing Xu, Shenyue Jiang, Xiaojiao Han, Longhua Wu, Renying Zhuo, Wenmin Qiu

**Affiliations:** ^1^ State Key Laboratory of Tree Genetics and Breeding, Key Laboratory of Tree Breeding of Zhejiang Province, Research Institute of Subtropical Forestry, Chinese Academy of Forestry, Hangzhou, China; ^2^ Faculty of Forestry, Nanjing Forestry University, Nanjing, China; ^3^ Key Laboratory of Three Gorges Regional Plant Genetic & Germplasm Enhancement (CTGU)/Biotechnology Research Center, China Three Gorges University, Yichang, China; ^4^ Meicheng Office of Market Supervision Bureau of Jiande City, Jiande, Zhejiang, China; ^5^ Key Laboratory of Soil Environment and Pollution Remediation, Institute of Soil Science, Chinese Academy of Sciences, Nanjing, China

**Keywords:** SpSIZ1, SpABI5, Cd stress, ABA, *Sedum plumbizincicola*

## Abstract

*Sedum plumbizincicola* is a renowned hyperaccumulator of cadmium (Cd), possesses significant potential for eco-friendly phytoremediation of soil contaminated with Cd. Nevertheless, comprehension of the mechanisms underpinning its Cd stress response remains constrained, primarily due to the absence of a comprehensive genome sequence and an established genetic transformation system. In this study, we successfully identified a novel protein that specifically responds to Cd stress through early comparative iTRAQ proteome and transcriptome analyses under Cd stress conditions. To further investigate its structure, we employed AlphaFold, a powerful tool for protein structure prediction, and found that this newly identified protein shares a similar structure with *Arabidopsis* AtSIZ1. Therefore, we named it *Sedum plumbizincicola* SIZ1 (SpSIZ1). Our study revealed that SpSIZ1 plays a crucial role in positively regulating Cd tolerance through its coordination with SpABI5. Overexpression of SpSIZ1 significantly enhanced plant resistance to Cd stress and reduced Cd accumulation. Expression pattern analysis revealed higher levels of SpSIZ1 expression in roots compared to stems and leaves, with up-regulation under Cd stress induction. Importantly, overexpressing SpSIZ1 resulted in lower Cd translocation factors (Tfs) but maintained relatively constant Cd levels in roots under Cd stress, leading to enhanced Cd stress resistance in plants. Protein interaction analysis revealed that SpSIZ1 interacts with SpABI5, and the expression of genes responsive to abscisic acid (ABA) through SpABI5-dependent signaling was significantly up-regulated in SpSIZ1-overexpressing plants with Cd stress treatment. Collectively, our results illustrate that SpSIZ1 interacts with SpABI5, enhancing the expression of ABA downstream stress-related genes through SpABI5, thereby increasing Cd tolerance in plants.

## 1 Introduction

Cadmium (Cd), a highly toxic and easily mobile heavy metal, presents significant risks to both plants and human health, while also causing severe environmental pollution (Zhao et al., 2015). Presently, agricultural soils across the globe are significantly impacted by Cd contamination (Haider et al., 2021). On one hand, plants can assimilate Cd from the soil and translocate it to various tissues, thereby facilitating its incorporation into the food chain and adversely affecting human health (Clemens et al., 2013). On the other hand, Cd inhibits various physiological processes in plants, including photosynthesis, respiration, and water movement, leading to damage in plant metabolism (Rizwan et al., 2016). Moreover, Cd stress can induce DNA damage, ultimately resulting in plant mortality (Galati et al., 2023). Therefore, it is crucial to reduce and mitigate Cd-induced pollution for the sake of environmental protection and human well-being.

*Sedum plumbizincicola* (hyperaccumulation ecotype, HE) is a Cd hyperaccumulator that has been discovered in mining regions in Quzhou, China. It possesses the capacity to accumulate Cd levels exceeding 100 ppm or 0.01% in its shoot dry biomass, while exhibiting minimal growth impairment in soils contaminated with Cd. Because of these characteristics, it is considered an important genetic resource for studying how plants respond to Cd stress and for remediating Cd-contaminated environments (Wu et al., 2012; Zhu et al., 2022). Currently, several key genes related to Cd tolerance have been identified in HE. The Cation/H+ exchanger (CAX) family proteins play a pivotal role in regulating ion homeostasis within the biological membrane system. SpCAX2n and SpCAX2h, situated on the tonoplast, are instrumental in sequestering Cd within vacuoles and are engaged in the detoxification of Cd (Zhang et al., 2015). The Natural resistance-associated macrophage protein 6 (SpNRAMP6) shows significantly increased expression in leaves when exposed to Cd and is involved in Cd translocation and accumulation in aboveground tissues (Chen et al., 2017). Heavy metal transporting ATPase (HMA) proteins are transmembrane transport proteins that use ATP hydrolysis to transport heavy metal cations. SpHMA3, localized on the vacuolar membrane, plays a specific role in Cd detoxification by actively transporting Cd ions into vacuoles for sequestration, thereby aiding in the normal growth of seedlings in Cd-contaminated soil (Liu et al., 2017a). Additionally, it has been found that SpHMA1 is situated at the chloroplast envelope and serves to safeguard photosynthesis by inhibiting the accumulation of Cd within the chloroplast (Zhao et al., 2019). Currently, most of the Cd tolerance-associated proteins discovered in HE are transport-related proteins, but the molecular mechanisms responsible for Cd detoxification in Cd hyperaccumulating plants HE are still not well elucidated.

Post-translational modification (PTMs) represents essential regulatory mechanisms governing a multitude of cellular processes (Hendriks and Vertegaal, 2016). The process of Small ubiquitin-like modifier (SUMO) conjugation, termed SUMOylation and mediated by SIZ1, is central to the regulation of activity, localization and stability of a plethora of intracellular effectors in eukaryotic organisms. In the context of drought stress regulation, *Arabidopsis* AtSIZ1 modulates plant growth and water deficit response through the regulation of gene expression (Catala et al., 2007). Additionally, the *Pepper’s* CaDSIZ1 augments drought tolerance by stabilizing the transcription factor CaDRHB1, specifically the dehydration-responsive homeobox 1 (Joo et al., 2022). In relation to heavy metal tolerance regulation, AtSIZ1 serves as a negative modulator in aluminum resistance, mediating the STOP1-ALMT1 pathway (Xu et al., 2021). Furthermore, AtSIZ1 augments Cd tolerance via the glutathione-dependent phytochelatin synthesis pathway (Zheng et al., 2022). Pertaining to the regulation of growth and development, AtSIZ1 exerts a negative influence on shoot regeneration, partly through the repression of wound-induced developmental reprogramming (Coleman et al., 2020). The Apple’s MdSIZ1 promotes the SUMOylation of auxin response factors 8 (MdARF8), modulating the formation of lateral root (Zhang et al., 2021). These findings indicate that SIZ1 plays important roles in multiple species. However, the role of SpSIZ1 in Cd tolerance in hyperaccumulating HE remains uncertain.

Abscisic acid (ABA) is a crucial hormone in plants that regulates responses to abiotic stress. When plants encounter drought, salt or other abiotic stresses, they rapidly accumulate ABA, which activates stress responses. Conversely, when environmental conditions improve, ABA levels decrease to basal levels, promoting plant growth (Zhu, 2016). The homeostasis of ABA is maintained through a delicate balance of its biosynthesis, catabolism and transport pathways. All the components involved in ABA homeostasis form a complex and intricate regulatory network, working together to regulate ABA levels (Chen et al., 2020). Previous studies have demonstrated that exogenous ABA can modulate endogenous ABA levels, thereby regulating the expression of related proteins and leading to increased Cd accumulation and enhanced resistance in hyperaccumulating plants (Lu et al., 2020). Moreover, studies have revealed the involvement of ACID-INSENSITIVE5 (ABI5), a crucial signaling molecule in the abscisic acid (ABA) pathway, in the ABA-mediated inhibition of Cd accumulation in *Arabidopsis* (Zhang et al., 2019). These findings highlight the crucial role of the ABA signaling pathway in regulating plant Cd tolerance.

In this study, we identified a Cd stress-responsive protein, SpSIZ1, through early comparative iTRAQ proteome and transcriptome analyses (Zhu et al., 2022), and scrutinized the role of SpSIZ1 in plant responses to Cd stress. Our results demonstrate that SpSIZ1 plays a pivotal role in augmenting plant tolerance to Cd stress and in diminishing Cd accumulation. Utilizing protein interaction analysis, we identified a significant interaction between SpSIZ1 and SpABI5, which results in the upregulation of ABA-response stress-related genes via SpABI5-mediated signaling. This interaction delineates a critical link between SpSIZ1 and SpABI5 under Cd stress, laying a substantial theoretical groundwork for subsequent studies exploring the interplay between SUMOylation and the ABA signaling cascade. Significantly, elucidating the molecular regulatory mechanisms of *S. plumbizincicola* in environments contaminated with Cd can inform the development of more efficacious Cd remediation methodologies and environmental conservation strategies.

## 2 Materials and methods

### 2.1 Plant material and growth conditions

*S. plumbizincicola* (hyperaccumulation ecotype, HE) and *S. alfredii* (non-hyperaccumulation ecotype, NHE) were sourced from mining areas in Quzhou, China, and a tea plantation in Hangzhou, China, respectively (Wu et al., 2012; Zhu et al., 2022). Stem segments from both ecotypes were aseptically excised and propagated in Hoagland-Arnon solution, with pH meticulously maintained at 5.8, and the solution was refreshed twice a week to ensure nutrient consistency. Utilizing the Hoagland-Arnon solution (Arnon and Hoagland, 1940) facilitated a controlled environment for nutrient uptake, particularly to mitigate interaction between phosphate and cadmium that could influence Cd assimilation. Plants were cultivated in a controlled growth chamber with controlled environmental condition at about 16/8 h in light/dark regime, 26/20°C in air temperatures, approximate 600 μmol m^-2^ s^-1^ in photon flux density, and 75% in relative humidity.

### 2.2 Protein sequence alignment and structure prediction

A homologous sequence of *S. plumbizincicola* SIZ1(SpSIZ1) was identified through a BLAST search, comparing it with the protein sequence of *Arabidopsis* SIZ1. The structural configurations of SpSIZ1 and AtSIZ1 were predict using AlphaFold (https://www.alphafold.ebi.ac.uk), a sophisticated tool for protein structure prediction (Jumper et al., 2021; Varadi and Velankar, 2023). The identification and characterization of protein domains for both SpSIZ1 and AtSIZ1 were facilitated by the NCBI conserved domain database (https://www.ncbi.nlm.nih.gov/Structure/cdd/wrpsb.cgi) (Marchler-Bauer et al., 2015). Multiple sequence alignment for SpSIZ1 and AtSIZ1was executed with ClustalX software, with the aligned sequences exported as an msf file. The alignments were then visualized and analyzed using GeneDoc software (https://nrbsc.org/gfx/genedoc). To infer evolutionary relationships, a Neighbor-joining phylogenetic tree was constructed in MEGA 7, with 1000 bootstrap repetitions to evaluate the robustness of the tree topology.

### 2.3 Cloning of *SpSIZ1* and plant transformation

The coding sequence (CDS) of SpSIZ1 was amplified from the cDNA of the HE utilizing the specific primers GFP-SpSIZ1-F/R (**Supplementary Table S2**). Subsequently, the amplified product was then ligated into the pCAMBIA1302 vector to generate the 35S::SpSIZ1-GFP construct through homologous recombination. The recombination vectors were introduced into *Agrobacterium tumefaciens* strain EHA105 via electroporation. The transformed *Agrobacterium* was subsequently used to infect leaf tissues of the NHE, following a modified version of a previously reported method (Liu et al., 2017a), resulting in the successful acquisition of transgenic plants with enhanced *SpSIZ1* expression. The transformation process involved making an incision on the leaves of *NHE* using a sterile scalpel, infusing the wounds with *Agrobacterium* containing the 35S::SpSIZ1-GFP construct, and selecting for transformants on a medium supplemented with hygromycin. White, hygromycin resistant callus formed at the inoculation sites was then excised and transferred to a shoot elongation medium, allowing for the development of seedlings. Once the seedlings reach a suitable size, they were transferred to a rooting medium, from which fully developed plants were obtained.

### 2.4 Cd stress treatment

The Cd stress treatment was conducted by subjecting young shoots from both the NHE and the 35S::SpSIZ1-GFP overexpression lines to Hoagland-Arnon nutrient solution supplemented with varying concentrations of CdCl_2_. Initially, plants were cultured for three weeks, after which only those exhibiting robust healthy and reaching approximately 10 cm in were transplanted into Hoagland-Arnon nutrient solution containing three distant concentration of CdCl_2_: 0 μM (control), 20 μM and 50 μM. Following a 10 d exposure period, the plants were harvested, treated with a 20 mmol/L EDTA solution for 20 min to chelate residual Cd, and subsequently rinsed with ddH_2_O to remove any external Cd. The plants were then carefully dissected into root, stem, and leaf segments for subsequent analysis.

### 2.5 Gene expression of *SpSIZ1*

To assess the impact of Cd stress on *SpSIZ1* expression, uniform HE plants were allocated into two separate groups, each exposed to 50 μM CdCl_2_ for either 0 h (control) or 24 h. Subsequent to the stress treatment, entire plants were harvested for RNA extraction. To elucidate the expression level of *SpSIZ1*, total RNA was extracted from various tissues of both the NHE and the 35S::SpSIZ1-GFP overexpression lines, encompassing roots, stems, and leaves. qPCR was conducted using the Applied Biosystems 7500 Real-Time PCR system (Waltham, MA, USA) with the TB GREEN Premix Ex Taq kit (Takara, Kyoto, Japan) to quantify gene expression. The housekeeping gene *UBC9* served as a reference for data normalization, and all qPCR reactions were executed in triplicate. the relative expression levels of *SpSIZ1* were calculated using the 2^^(-ΔΔCt)^ method (Livak and Schmittgen, 2001), to facilitate the comparison of expression levels under different conditions. Primer sequences utilized for qPCR are detailed in **Supplementary Table S2**, and all experiments were repeated with three biological replicates to ensure reliability.

### 2.6 Expression of SpSIZ1 protein in NHE and 35S::SpSIZ1-GFP lines

Total protein extraction was performed on whole plants from both the NHE and the 35S::SpSIZ1-GFP overexpression lines, encompassing roots, stems, and leaves, for subsequent immunoblot analysis. The detection of the SpSIZ1-GFP fusion protein was facilitated using a monoclonal anti-GFP antibody (ab290, Abcam), while Ponceau S staining was utilized as a loading control to ensure equal protein loading.

### 2.7 Physiological analysis

Uniform-sized stem segments from both the NHE and 35S::SpSIZ1-GFP lines were cultured in a hydroponic system. After a two week growth period, plants of uniform size were selected for analysis, with their initial fresh weight (W1) recorded prior to Cd stress exposure. The plants were then subjected to Cd stress treatment with 20 μM and 50 μM CdCl_2_ over a 10 d period, with a 0 μM CdCl_2_ treatment serving as the negative control. Post-treatment, the fresh weight (W2) was reassessed uniformly for comparison. The maximal PSII quantum yield, indicated by the Fv/Fm ratio was determined using a pulse modulation fluorometer (MINI-PAM, Heinz Walz, Germany) as previously described (Zhu et al., 2011). To quantify the Malondialdehyde (MDA) content as a marker of lipid peroxidation, consistent growth materials from both NHE and 35S::SpSIZ1-GFP overexpression lines were treated with either 0 μM or 50 μM CdCl_2_ for 10 d. An equal amount of these material was ground to a homogenized state for the subsequent thiobarbituric (TBA) assay (Zhang et al., 2023). For each plant line, three biological replicates were measured to ensure the reliability of the results. Representative phenotypic images were captured 10 d after the application of 50 μM CdCl_2_ to document the morphological responses to stress.

### 2.8 Subcellular localization analysis

The CDS of SpSIZ1 was amplified from the cDNA of the HE using the specific primers GFP-105-F/R (**Supplementary Table S2**). Subsequently, the amplified product was then ligated into the pCAMBIA1302-GFP vector to create the pCAMBIA1302-GFP-SpSIZ1 vector construct via homologous recombination. The plasmids were transferred into the protoplasts of the HE. GFP empty vector serves as a negative control. The protoplasts were subsequently cultured for a duration of 18 h and visualized using a Leica/TCS SP8 confocal microscope under the following conditions: GFP fluorescence was excited at 488 nm using a 63× oil objective (Asai et al., 2018).

### 2.9 Determination of Cd content in plant

The uniform NHE and 35S::SpSIZ1-GFP lines were treated with 50 μM CdCl_2_ for 10 d, with 0 μM CdCl_2_ serving as the negative control. After the Cd stress treatment, the roots of each treated genotype were rinsed with ddH_2_O for three times. Roots, stems, and leaves tissues from each plant were collected separately and dried to a constant weight in a 65°C oven. The dried tissues were sent to the National Forestry and Grassland Administration Economic Forest Product Quality Inspection and Testing Center (Hangzhou) for determination of Cd content. Each sample was prepared with three biological replicates, with the NHE used as a control.

### 2.10 Yeast two-hybrid assays

The CDS of SpSIZ1 was amplified from the cDNA of the HE using the specific primers BD-F/R and cloned into the pGBKT7 (BD) vector, while the CDS of SpAIB5 was amplified from the cDNA of the HE using the specific primers AD-F/R (**Supplementary Table S2**) and cloned into the pGADT7 (AD). These constructs were subsequently introduced into the yeast strain Y2H Gold, following the protocol (Clontech, 630489). The yeast two-hybrid (Y2H) assay was performed according to a previously described method (Jiang et al., 2019). Each assay was repeated three times and yielded consistent results.

### 2.11 Co-immunoprecipitation in HEK293T cells

Human embryonic kidney (HEK) 293T cells were cultured in Dulbecco’s modified Eagle’s medium (DMEM). The CDS of SpSIZ1 and SpABI5 was amplified from the cDNA of the HE using the specific primers (**Supplementary Table S2**). GFP-SpSIZ1 and Myc-SpABI5 proteins were co-expressed in HEK293T cells and co-immunoprecipitation was performed in HEK293T cells with slight modifications based on previous reports (Liu et al., 2017b). Specifically, GFP-trap beads (ChromoTek GFP-Trap^®^ Magnetic Agarose) were used for specific enrichment of the GFP-tagged protein. The proteins that were attached to the beads were released using 2×SDS Sample Buffer through heating at 100°C for 8 min, followed by analysis using immunoblotting (Liu et al., 2020). GFP antibodies (1:3000 dilution, ab290, Abcam) were employed for immunoprecipitation signal collection, while Myc antibodies (1:2000 dilution, M047-3, MBL) were used to capture the interaction signals.

### 2.12 Luciferase complementation imaging assay

The CDS of SpSIZ1 and SpABI5 was amplified from the cDNA of the HE using the specific primers (**Supplementary Table S2**) and inserted in-frame and upstream of the sequence that encodes the N-terminal half of firefly luciferase (nLUC) or the C-terminal half of luciferase (cLUC), respectively. The SpSIZ5-nLUC and SpABI5-cLUC constructs were separately transformed into *Agrobacterium* GV3101, followed by equimolar infiltration into the leaves of 3-week-old *Nicotiana benthamiana*, allowing for the recombinant DNA to be delivered into plant cells and stably expressed. After 2 d, LUC signals were detected by infiltrating the leaves with a solution containing 0.5 mM luciferin and capturing images using a TANON Chemiluminescent Imaging system.

### 2.13 Bimolecular fluorescence complementation assay

The CDS of SpSIZ1 and SpABI5 was amplified from the cDNA of the HE using the specific primers (**Supplementary Table S2**) and were inserted into cYFP and nYFP, respectively. This generated constructs with N-terminal fusions of SpSIZ1 and C-terminal fusions of SpABI5. The BiFC were performed by bombarding these constructs into HE protoplasts, then the protoplasts were cultured for 18 h and photographed through a Leica/TCS SP8 confocal microscope (Leica Microsystems, Wetzlar, Germany) with the following conditions: GFP, 488 nm, 63× oil objective (Asai et al., 2018).

### 2.14 Statistical analysis

All statistical data were analyzed using GraphPad Prism 8.0.2 software. Statistical differences were evaluated using ANOVA with a two-tailed Student’s t-test. The significance levels were denoted as follows: ns for *p*-value > 0.05, * for *p*-value < 0.05, ** for *p*-value < 0.01, and *** for *p*-value < 0.001 (Zou et al., 2022).

## 3 Results

### 3.1 Overexpression of SpSIZ1 does not affect plant growth

**Figure 1:**
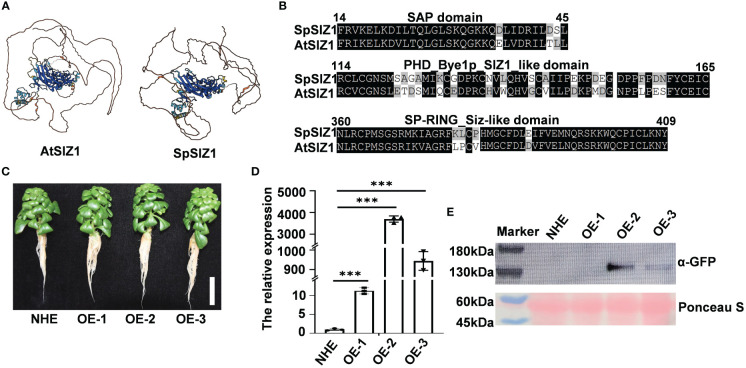
Identification of SpSIZ1. **(A)** Protein structure alignment of AtSIZ1 and SpSIZ1 using AlphaFold. Regions with a model confidence per-residue Local-Distance Difference Test (pLDDT) score greater than 70 are highlighted, indicating high similarity between the two proteins. **(B)** Protein domain similarity analysis of AtSIZ1 and SpSIZ1. Conserved domains, including the SAP domain, PHD domain, and Sp-RING finger domain, are indicated. **(C)** Phenotypes of the wild type (WT) NHE and SaSIZ1-OE lines (SpSIZ1-OE-1, SpSIZ1-OE-2, and SpSIZ1-OE-3) at 14 d after transplanting into the soil. Scale bar: 3 cm. **(D)** Relative *SpSIZ1* transcript levels in the NHE, SpSIZ1-OE-1, SpSIZ1-OE-2, and SpSIZ1-OE-3. The data were normalized to the expression level in NHE, which was assigned a value of 1. **(E)** Detection of SpSIZ1-GFP protein accumulation in the NHE, SpSIZ1-OE-1, SpSIZ1-OE-2, and SpSIZ1-OE-3 using an anti-GFP antibody. Ponceau S staining was used as a loading control. *** for *p*-value < 0.001.

The identified SpSIZ1 gene consists of a 2586 bp coding sequence (CDS) encoding 862 amino acids in *Sedum plumbizincicola* (**Supplementary Figure S1**). Subsequently, we employed AlphaFold to predict the protein structures of SpSIZ1. We observed that regions with a model confidence per-residue Local-Distance Difference Test (pLDDT) score greater than 70 exhibited high similarity, indicating a strong homology between SpSIZ1 and AtSIZ1 proteins (**Figure 1A**). Furthermore, analysis of conserved domains revealed that SpSIZ1 contains three domains, including an SAP domain (involved in DNA binding in yeast), a PHD domain (participating in ligase catalytic substrate connection), and a Sp-RING finger domain (providing catalytic function to the AtSIZ1 ligase) (**Figures 1B**, **Supplementary Figure S1**).

To explore the physiological function of SpSIZ1, we obtained overexpression lines of SpSIZ1 designated as SpSIZ1-OE-1, SpSIZ1-OE-2, and SpSIZ1-OE-3, respectively. Comparative analysis with the wild-type NHE revealed that the overexpression lines of SpSIZ1 did not show any impact on plant growth (**Figure 1C**). Our results revealed that the SpSIZ1-OE-2 line displayed the highest expression level, whereas the SpSIZ1-OE-1 line exhibited the lowest expression level (**Figures 1D, E**).

### 3.2 SpSIZ1 positively regulates plant Cd tolerance

**Figure 2:**
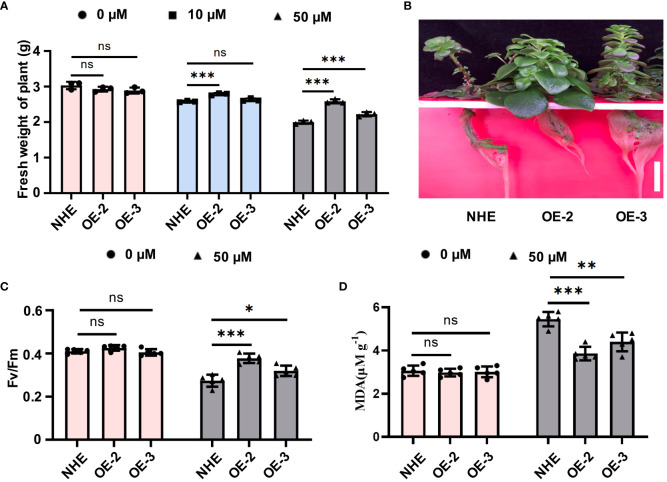
The Cd stress tolerance of SpSIZ1-OE lines significantly improves compared to WT NHE. **(A)** The fresh weight of NHE, SpSIZ1-OE-1, and SpSIZ1-OE-2 was measured after 10 d of different Cd stress treatments. **(B)** The phenotypes of NHE, SpSIZ1-OE-2, and SpSIZ1-OE-3 were observed after 10 d of 50 μM CdCl_2_ stress treatment. Scale bar: 3 cm. The Fv/Fm **(C)** and MDA content **(D)** values of NHE, SpSIZ1-OE-1, and SpSIZ1-OE-2 were assessed after 10 d of 50 μM CdCl_2_ stress treatment. ns for *p*-value > 0.05, * for *p*-value < 0.05, ** for *p*-value < 0.01, and *** for *p*-value < 0.001.

To ensure consistent growth among different plants, both the NHE and SpSIZ1-OE lines were simultaneously cultivated in a hydroponic system without CdCl_2_ for two weeks (**Supplementary Figure S2**). To evaluate the role of SpSIZ1 in Cd tolerance response, both the NHE and SpSIZ1-OE lines were subjected to treatments of 0 µM CdCl_2_ (no CdCl_2_), 20 µM CdCl_2_ (low concentration), and 50 µM CdCl_2_ (high concentration) for 10 d (**Supplementary Figure S3**). Under the 0 µM CdCl_2_ treatment, both the NHE and SpSIZ1-OE lines exhibited normal growth. Under the 20 µM CdCl_2_ treatment, both lines showed slight growth inhibition, but SpSIZ1-OE2 exhibited weaker growth inhibition compared to the NHE, resulting in higher fresh weight. Under the 50 µM CdCl_2_ treatment, both lines experienced severe growth inhibition. However, SpSIZ1-OE-2 and SpSIZ1-OE-3 showed weaker growth inhibition compared to the NHE, leading to higher fresh weight (**Figure 2A**). Moreover, compared to the NHE, both SpSIZ1-OE-2 and SpSIZ1-OE-3 exhibited typical Cd tolerance characteristics, such as normal leaf morphology without shedding and normal root growth. Additionally, SpSIZ1-OE-2 showed significantly higher resistance than SpSIZ1-OE-3, which is consistent with its higher mRNA and protein expression levels (**Figure 2B**). Both Fv/Fm and MDA content are important indicators for assessing the extent of plant damage. Under the 50 µM CdCl_2_ treatment, both SpSIZ1-OE-2 and SpSIZ1-OE-3 exhibited higher Fv/Fm values and lower MDA content values compared to the NHE, indicating that the SpSIZ1-OE lines suffered less damage (**Figures 2C, D**). These results collectively demonstrate that *SpSIZ1* positively regulates plant Cd tolerance in the NHE.

**Figure 3:**
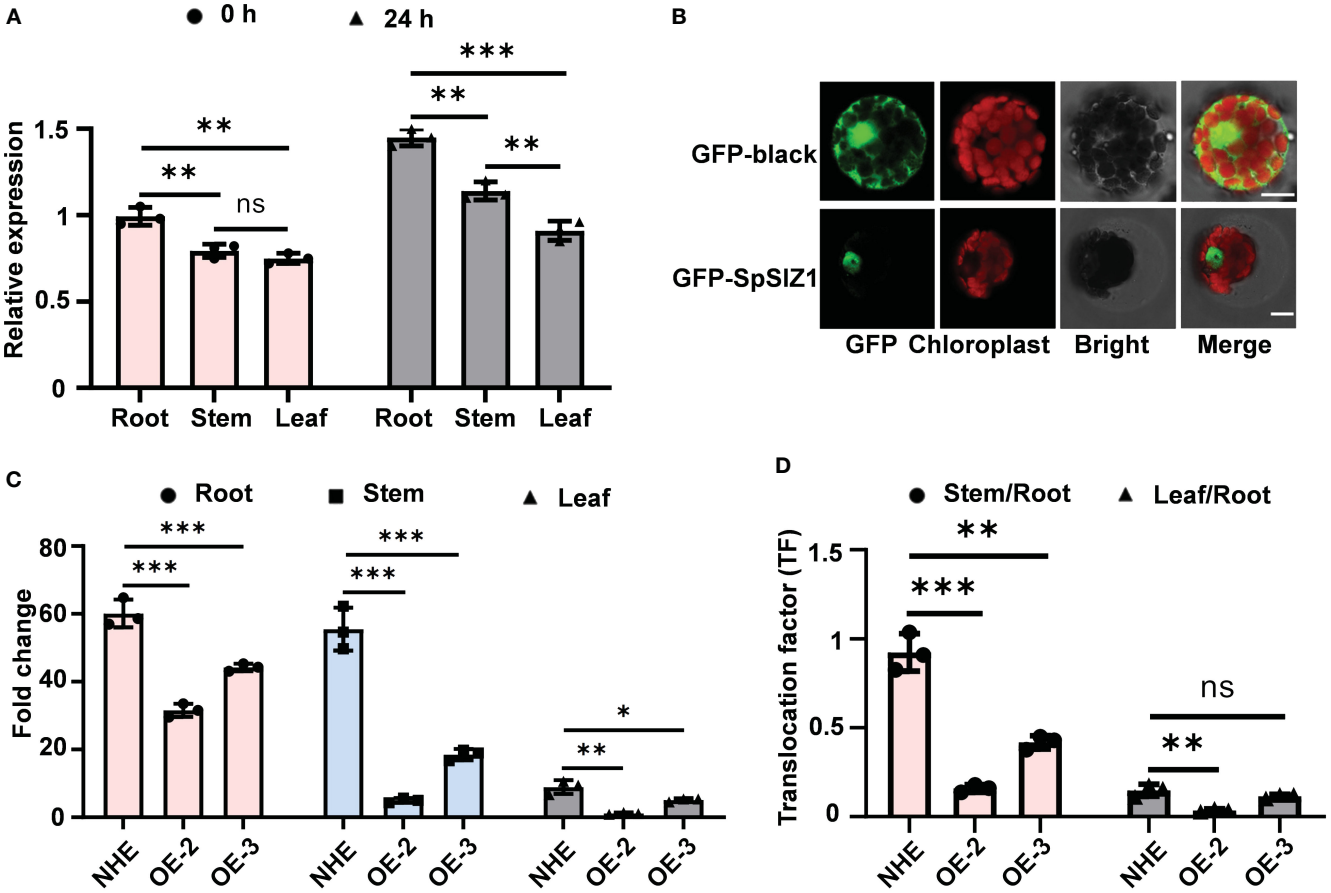
SpSIZ1 affects Cd accumulation. **(A)** Expression patterns of *SpSIZ1* before and after 50 μM CdCl_2_ stress treatment for 24 h were analyzed. **(B)** Subcellular localization analysis of GFP-black and GFP-SpSIZ1 in HE protoplasts. Scale bar: 10 µm. **(C)** The SpSIZ1-OE lines exhibited reduced Cd accumulation in the root, stem, and leaf under 50 μM CdCl_2_ stress treatment for 10 d compared to the wild-type NHE. **(D)** Translocation factors (TFs) significantly decreased in SpSIZ1-OE lines under 50 μM CdCl_2_ stress treatment for 10 d compared to the wild-type NHE. Data are presented as mean ± SD (n = 3). ns for *p*-value > 0.05, * for *p*-value < 0.05, ** for *p*-value < 0.01, and *** for *p*-value < 0.001.

### 3.3 SpSIZ1 significantly reduced plant Cd accumulation

To gain further insights into the mechanism underlying SpSIZ1-mediated Cd tolerance, gene expression analysis was performed. Under no CdCl_2_ treatment, *SpSIZ1* was expressed in various tissues, with the highest level observed in the root. However, under CdCl_2_ treatment, its expression was induced and up-regulated in all tissues (**Figure 3A**). Subcellular localization analysis using SpSIZ1-GFP fusion protein in HE protoplasts revealed that the GFP signal from the empty GFP vector was distributed throughout the entire cell, while the GFP signal from SpSIZ1-GFP was exclusively localized in the nucleus, indicating that SpSIZ1 specifically localizes to the nucleus (**Figure 3B**).

To determine the role of SpSIZ1 in Cd accumulation, Cd contents in various tissues of WT and SpSIZ1-OE lines were examined. Under the treatment of 50 µM CdCl_2_, significant increases in Cd accumulation were observed in the root, stem, and leaf of NHE. In contrast, the SpSIZ1-OE-2 line exhibited a comparatively lower increase in Cd accumulation, while the SpSIZ1-OE-3 line displayed intermediate levels of Cd accumulation in the root, stem, and leaf. These results indicated that the SpSIZ1-OE lines exhibited reduced plant Cd accumulation compared to NHE, with SpSIZ1-OE-2 showing lower Cd accumulation than SpSIZ1-OE-3 (**Figure 3C**, **Supplementary Table S1**). Furthermore, under CdCl_2_ treatment, the translocation factors (TFs) in the SpSIZ1-OE lines was significantly decreased compared to NHE, suggesting that the SpSIZ1-OE lines either do not absorb Cd or do not transport it to the aboveground parts (**Figure 3D**).

### 3.4 SpSIZ1 interacts with SpABI5 both *in vitro* and *in vivo*

**Figure 4:**
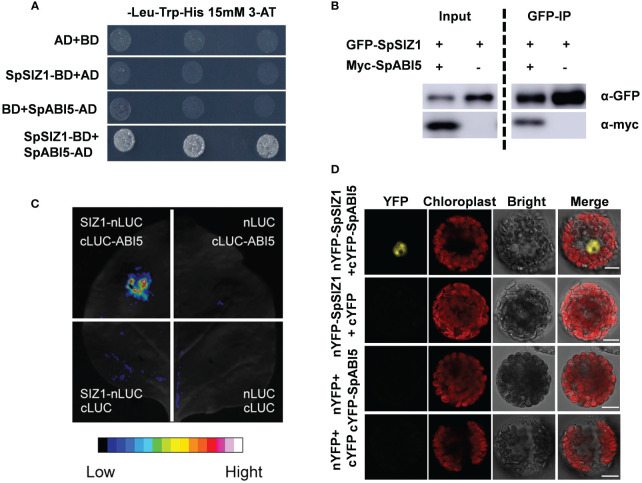
SpSIZ1 interacts with SpABI5 *in vitro* and *in vivo*. **(A)** Y2H assay demonstrates the interaction between SpSIZ1 and SpABI5 under 15 mM 3-AT treatment. **(B)** Co-IP assay confirms the interaction between SpSIZ1 and SpABI5 in HEK293T cells. GFP-SpSIZ1 was immunoprecipitated using GFP-trap beads, and SpSIZ1-interacting proteins were detected by immunoblotting with an α-myc antibody. **(C)** LCI assay shows the interaction between SpSIZ1 and SpABI5 in *Nicotiana benthamiana* leaves infiltrated with the indicated constructs. **(D)** BiFC assays demonstrate that SpSIZ1 and SpABI5 interact in HE protoplasts. Scale bar: 10 µm.

The above results indicate that SpSIZ1 plays a positive role in regulating plant Cd tolerance. To further explore the potential regulators involved in SpSIZ1-mediated Cd tolerance, we conducted a yeast two-hybrid screening and identified the basic region/Leu zipper transcription factor ABSCISIC ACID-INSENSITIVE5 homologue protein (SpABI5) as an interacting partner of SpSIZ1. The interaction between SpSIZ1 and SpABI5 was confirmed through various assays. Firstly, we performed protein-protein interaction analysis *in vitro*. The SpSIZ1 protein was fused to the pGBKT7 vector (BD-SpSIZ1), and the SpABI5 protein was introduced into the pGADT7 vector (AD-SpABI5). The Y2H assay showed that SpSIZ1 interacted with SpABI5 in yeast cells grown on selective medium lacking Leu, Trp, and His (**Figure 4A**). Additionally, we co-cultured GFP-SpSIZ1 and Myc-SpABI5 in HEK293T cells and validated the interaction between SpSIZ1 and SpABI5 through Co-immunoprecipitation (**Figure 4B**). Furthermore, the interaction between SpSIZ1 and SpABI5 was confirmed using the LCI (LUC complementation imaging) technique. Only in tobacco leaves co-infiltrated with SpSIZ1-nLuc and SpABI5-cLuc constructs, luciferase signals were observed, indicating the physical interaction between SpSIZ1 and SpABI5 (**Figure 4C**). Secondly, we performed protein-protein interaction analysis *in vivo* using the BiFC (bimolecular fluorescence complementation) assay in HE protoplasts. When SpSIZ1-nYFP and SpABI5-cYFP were co-transfected into HE protoplasts, strong yellow fluorescent protein (YFP) signals were observed in the nuclei, indicating that SpSIZ1 and SpABI5 co-localize and interact in the nucleus (**Figure 4D**). Taken together, these data confirm that SpSIZ1 interacts with SpABI5 both *in vitro* and *in vivo*, suggesting a potential role for SpABI5 in mediating the Cd tolerance regulated by SpSIZ1.

### 3.5 The interaction of SpSIZ1 and SpABI5 enhances the expression levels of ABA-related genes

**Figure 5:**
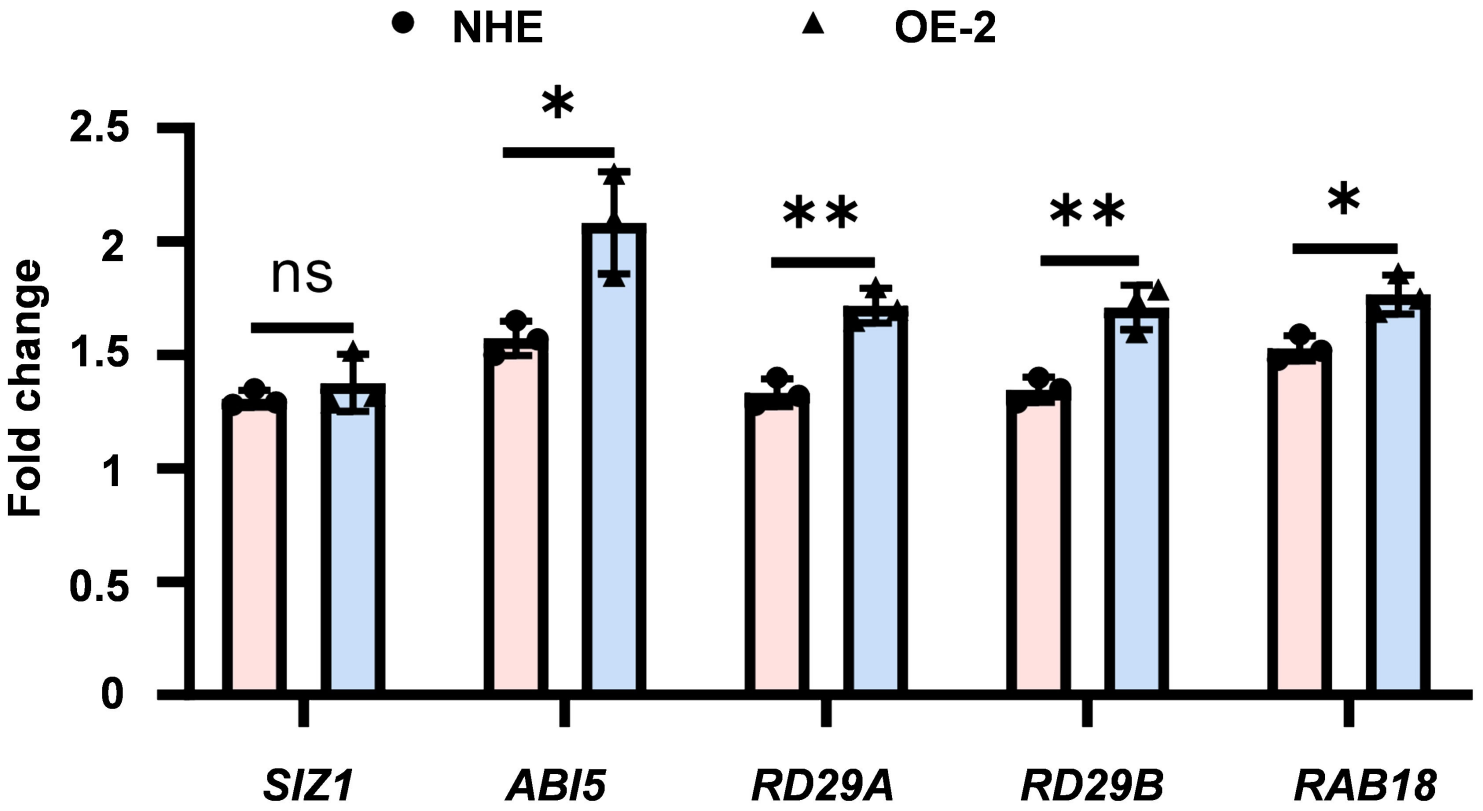
Expression of ABA-related genes under 50 μM CdCl_2_ stress treatment for 10 d. The fold change was normalized to the expression level under 0 μM CdCl_2_ stress treatment for 10 d, which was set to 1. Data are presented as mean ± SD (n = 3). ns for *p*-value > 0.05, * for *p*-value < 0.05, and ** for *p*-value < 0.01.

ABI5, a crucial transcription factor in the ABA signaling pathway, plays a vital role in plant stress tolerance. The expression level of ABI5 and downstream stress-responsive genes is important for plant tolerance to various stresses (**Figure 5**). Considering that SpSIZ1 interacts with SpABI5 to regulate plant Cd tolerance, we examined the expression of *SpABI5* and downstream stress-responsive genes (*RD29A*, *RD29B*, and *RAB18*) under CdCl_2_ treatment in both NHE and SpSIZ1-OE lines. Firstly, under CdCl_2_ treatment, both NHE and SpSIZ1-OE lines showed induced and up-regulated expression of *SpSIZ1*. However, there was no significant difference in the fold change of up-regulation between the two lines. Similarly, *SpABI5* was also induced and up-regulated, but the fold change in SpSIZ1-OE lines was significantly higher than in NHE. This result indicates that under CdCl_2_ treatment, the interaction between SpSIZ1 and SpABI5 indeed regulates the expression level of endogenous *SpABI5*. Secondly, the expression levels of ABA downstream stress-responsive genes (*RD29A*, *RD29B*, and *RAB18*) were validated in both NHE and SpSIZ1-OE lines under CdCl_2_ treatment. The results showed that *RD29A*, *RD29B*, and *RAB18* were significantly up-regulated in both NHE and SpSIZ1-OE lines. However, the fold change of regulation in the SpSIZ1-OE line was significantly higher than in NHE. This indicates that the interaction between SpSIZ1 and SpABI5 enhances the expression levels of ABA-related genes, thereby regulating plant Cd tolerance.

## 4 Discussion

### 4.1 Protein structure similarity analysis provides clues for studying the function of SpSIZ1

Protein sequence similarity alignment is a crucial method for studying the function of homologous proteins. The widely used approach for this purpose is the sequence similarity-based BLAST alignment tool (Kent, 2002; González-Pech et al., 2019; Hu and Kurgan, 2019), which aims to identify homologous sequences and infer various characteristics of the query sequence, including function, structure, and co-evolution. However, sequence-based homology inference has limitations in detecting distant evolutionary relationships solely based on sequence similarity. To overcome this limitation, protein domain prediction can be employed to assess whether aligned homologous proteins share the same domains, thereby inferring potential functional similarities (Marchler-Bauer et al., 2015; Yang et al., 2020). Additionally, detecting similarities between protein structures in 3D space provides higher sensitivity in identifying homologous proteins. Recent advancements in artificial intelligence have made accurate prediction of the 3D structures of unknown proteins and comparison of structural similarities between known proteins possible. The groundbreaking AlphaFold algorithm is a notable development in this field (Jumper et al., 2021; van Kempen et al., 2023; Varadi and Velankar, 2023). In our study, we initially used a sequence similarity-based BLAST search to identify the homologous protein SpSIZ1 in HE to AtSIZ1. Subsequently, protein domain prediction revealed that both SpSIZ1 and AtSIZ1 possess the same domains. Finally, the AlphaFold algorithm was utilized to predict the 3D spatial structures of SpSIZ1 and AtSIZ1 proteins. The combined results from these three methods strongly indicate a high degree of homology between SpSIZ1 and AtSIZ1 proteins. Through this structural comparison, we can infer that SpSIZ1 may have similar functions to AtSIZ1 in the Cd stress pathway, providing important clues for studying how *SpSIZ1* is involved in Cd tolerance in *Sedum plumbizincicola*. The results above indicated the important role of AlphaFold in predicting protein structures, but further physiological and biochemical experiments are needed to validate the specific protein functions.

### 4.2 *SpSIZ1* perform the function of positively regulating plant Cd tolerance

*SIZ1* is known to participate in multiple physiological processes, such as cell division, photomorphogenesis, flowering, hormone signaling, and responses to biotic and abiotic stresses. It can function as both a positive and negative regulator. In *Arabidopsis siz1* mutants, there is a significant increase in salicylic acid (SA) content, leading to elevated expression of pathogenesis-related genes. AtSIZ1 acts as a negative regulator of SA-mediated immune signaling (Lee et al., 2007). In *pepper*, CaDSIZ1 positively regulates drought tolerance by stabilizing the transcription factor CaDRHB1 (Joo et al., 2022). However, in *Arabidopsis*, AtSIZ1 exhibits dual regulation of drought resistance, which may be influenced by different environmental conditions or detection indicators (Catala et al., 2007; Miura et al., 2013). Moreover, AtSIZ1 also contributes to enhanced resistance against aluminum and Cd stress, positively regulating responses to these metal stresses (Xu et al., 2021; Zheng et al., 2022). These findings suggest that SIZ1 plays a role in modulating various stress responses, although the regulatory patterns may vary. Further research is required to comprehend the specific functions of SIZ1 in different species and under diverse stress conditions. In our experiments, we observed that SpSIZ1 and AtSIZ1 share the same function and can positively regulate plant Cd tolerance. Overexpression of *SpSIZ1* significantly enhances Cd stress resistance in NHE, manifested by normal growth and development of Cd-stressed plants, as well as normal physiological and biochemical indicators (Fv/Fm and MDA content) along with significant changes in the expression of related genes *in vivo*. However, due to the lack of genetic materials and experimental methods, the specific molecular mechanisms underlying the involvement of *SpSIZ1* in Cd stress tolerance remain unclear.

### 4.3 SpSIZ1 effectively decreased the accumulation of Cd in NHE plants

The accumulation of Cd can induce varying levels of toxicity in plants, leading to the activation of different defense signaling pathways. Changes in gene expression play a crucial role in regulating plant tolerance to Cd stress (Rizwan et al., 2016). A heavy metal transport protein gene called SpHMA3 from HE was cloned, which exhibits Cd hyper-tolerance. Interestingly, there was no significant difference in Cd concentration between wild-type and transgenic plants, indicating that Cd hyper-accumulation and hyper-tolerance are independent traits in HE (Liu et al., 2017a). In our research, we identified *SpSIZ1* as a key gene involved in Cd detoxification and found that it positively regulates Cd tolerance. *SpSIZ1* plays a vital role in maintaining normal growth and development of young leaves in the presence of Cd. Moreover, *SpSIZ1* effectively reduces Cd accumulation in NHE. These findings suggest that *SpSIZ1* mediates Cd tolerance in HE but does not contribute to the high Cd accumulation observed. Therefore, *SpSIZ1* may play a role in reducing Cd accumulation in HE. In the future, the use of gene editing technology to directly knock out the *SpSIZ1* gene in HE can help clarify the role of *SpSIZ1* in Cd accumulation. This approach will contribute to elucidating the relationship between the *SpSIZ1* and the characteristic of Cd hyperaccumulation in HE, resolving any potential contradictions that may exist. Since SpSIZ1 is localized in the nucleus, it may function as a signaling molecule, participating in both Cd accumulation and tolerance as independent traits. However, further investigations are needed to elucidate the specific mechanisms involved. In addition, related studies have found that the interaction between phosphate and Cd can lead to reduced Cd accumulation in rice (Chen et al., 2022). In our experiment, we used the standard Hoagland-Arnon solution (Arnon and Hoagland, 1940) and strictly controlled the consistency of phosphate concentration in the nutrient solution. However, it is unknown whether the *SpSIZ1* gene is involved in the interaction between phosphate and Cd, which needs to be further elucidated in future studies. In addition, *SIZ1* also plays a major role in phosphate starvation response (PSR) as it sumoylates PHOSPHATE STARVATION RESPONSE1(PHR1) (Miura et al., 2005), the master regulator of PSR genes. Therefore, more evidence is needed to demonstrate whether there is a relationship between phosphate, Cd and *SpSIZ1*.

### 4.4 The interaction between SpSIZ1 and SpABI5 is involved in plant Cd tolerance

**Figure 6:**
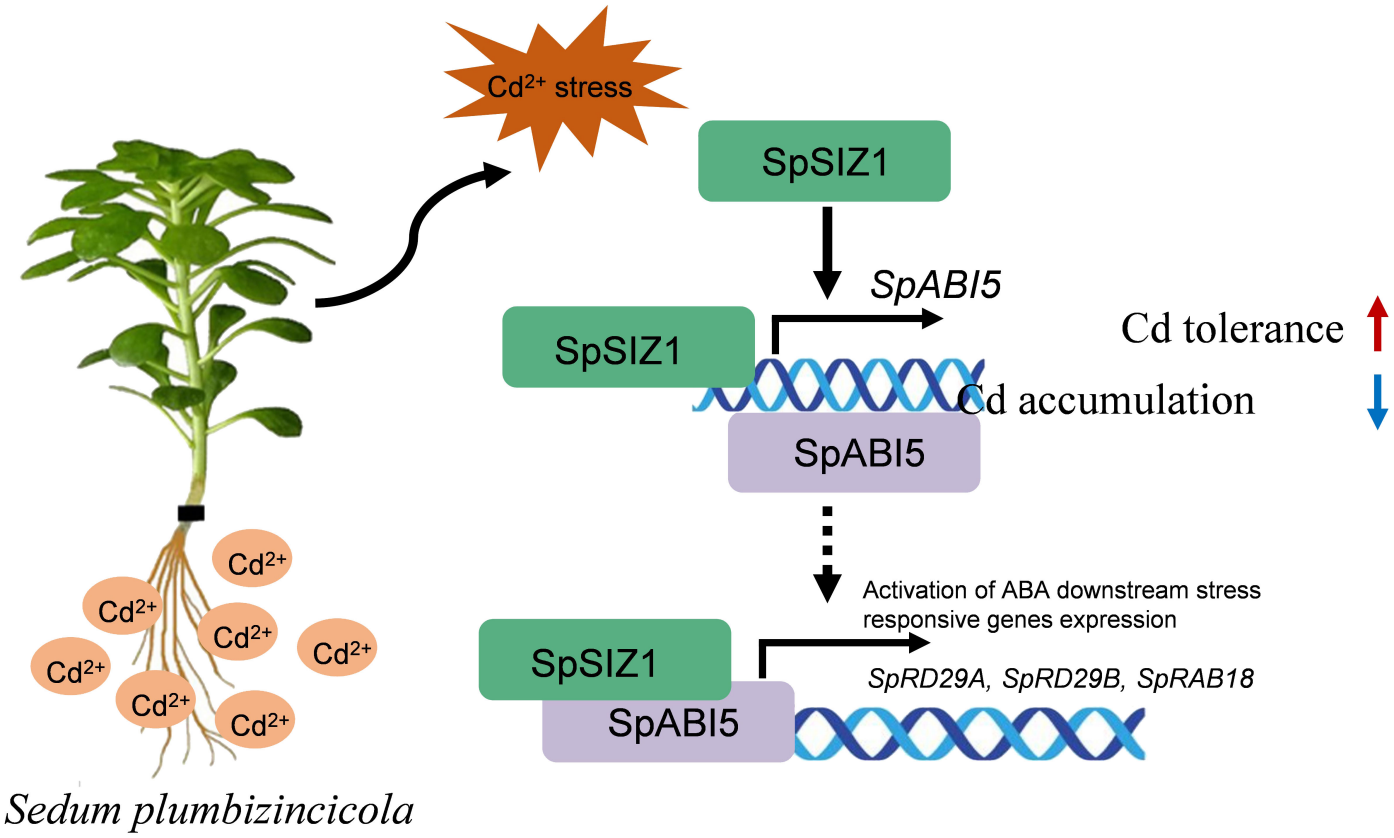
Proposed model for Cd tolerance orchestrated by the SpSIZ1–SpABI5 module.

SIZ1-mediated SUMOylation and ABA5-mediated ABA signaling have been shown to play roles in various biotic and abiotic stresses (Hendriks and Vertegaal, 2016; Zhu, 2016). This study discovered that SpSIZ1 and SpABI5 interact with each other and regulate plant Cd tolerance, establishing a significant connection between the ABA signaling pathway and Cd tolerance pathway. Previous studies have demonstrated that SIZ1-mediated SUMOylation modifies substrates and regulates the expression of related proteins (Hendriks et al., 2017). However, due to the challenges in genetic transformation of HE, this study only obtained materials related to SpSIZ1 and not SpABI5. Therefore, it remains unknown whether SpSIZ1 can SUMOylate SpABI5. Additionally, this study found that the interaction between SpSIZ1 and SpABI5 leads to the up-regulation of *ABI5* and downstream stress-related genes, thereby regulating plant Cd tolerance. *ABI5*, as a key gene in the ABA signaling pathway, has been found to regulate plant responses to various stresses (Wang et al., 2021; Liu et al., 2023). However, further validation is required to determine whether SpABI5 directly participates in the regulation of plant Cd tolerance. In summary, we propose a model for the regulation of Cd stress by SpSIZ1, in which SpSIZ1 enhances plant tolerance to Cd stress through SpABI5-mediated signaling and up-regulates *SpABI5* expression under Cd stress (**Figure 6**). Simultaneously SpSIZ1 interacts with SpABI5 to enhance its transcriptional activation activity towards target genes (**Figure 6**). Understanding the molecular regulatory mechanisms of *Sedum plumbizincicola* in Cd-contaminated environments not only enhances our knowledge of plant responses to Cd stress but also provides valuable insights for the development of advanced Cd remediation techniques and environmental protection strategies. By exploring the intricate pathways and genetic factors involved in Cd hyperaccumulation and tolerance, we can potentially utilize the unique properties of *Sedum plumbizincicola* to engineer plants with enhanced Cd uptake and sequestration capabilities, thereby facilitating the restoration of Cd-contaminated soils and safeguarding ecosystems.

## Data availability statement

The original contributions presented in the study are included in the article/**Supplementary Material**. Further inquiries can be directed to the corresponding authors.

## Author contributions

YL: Conceptualization, Data curation, Formal analysis, Software, Validation, Visualization, Writing – original draft, Writing – review & editing. ZH: Formal analysis, Funding acquisition, Visualization, Writing – original draft. JX: Formal analysis, Investigation, Supervision, Validation, Visualization, Writing – review & editing. SJ: Formal analysis, Resources, Software, Visualization, Writing – review & editing. XH: Formal analysis, Software, Visualization, Writing – review & editing. LW: Formal analysis, Supervision, Writing – review & editing. RZ: Funding acquisition, Investigation, Supervision, Visualization, Writing – review & editing. WQ: Formal analysis, Funding acquisition, Investigation, Validation, Writing – original draft, Writing – review & editing.
