# Supplementary material for: SpSIZ1 from hyperaccumulator Sedum plumbizincicola orchestrates SpABI5 to fine-tune cadmium tolerance
Source: Front Plant Sci. 2024 Jul 8;15:1382121. doi: 10.3389/fpls.2024.1382121 (PMC11264288; doi:10.3389/fpls.2024.1382121)
Supplement: Supplementary file 1 [file DataSheet_1.docx]

**Supplemental figures**


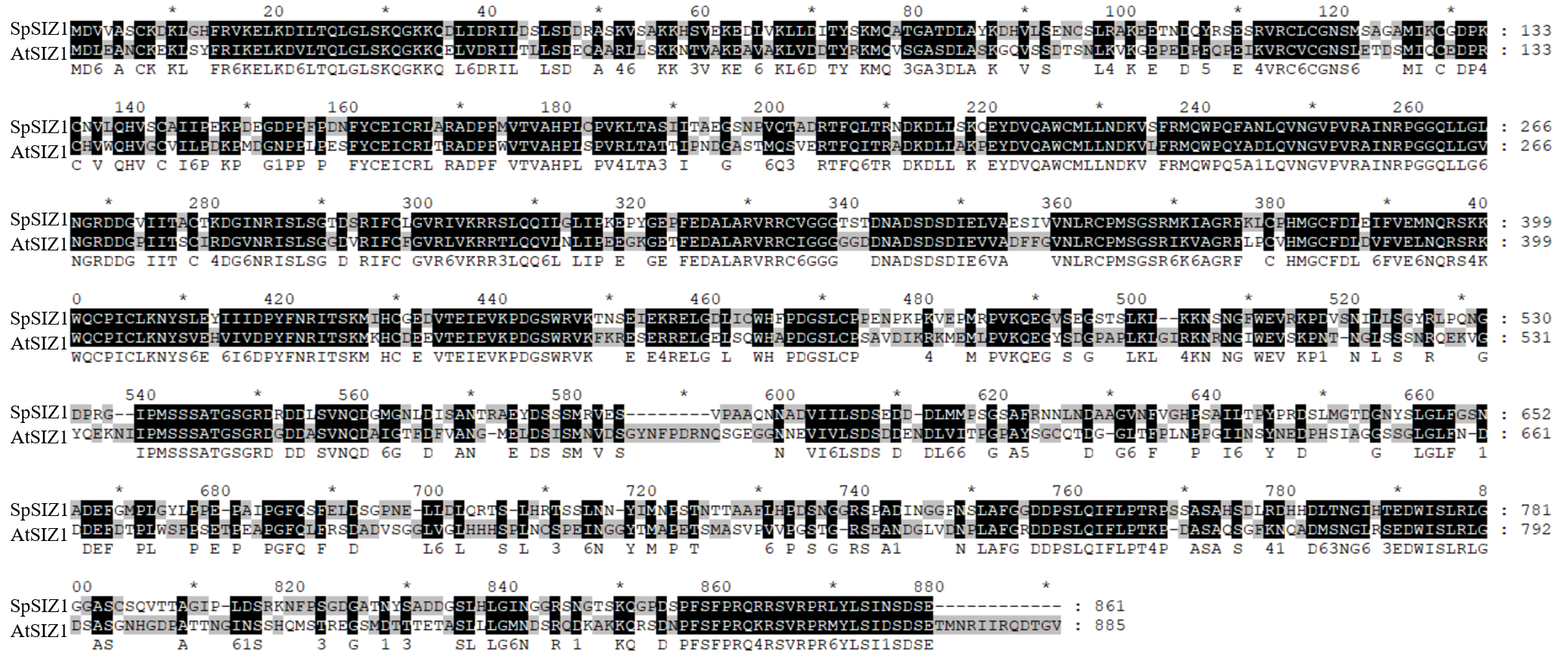


**Figure S1.** Full-length protein structure similarity alignment of AtSIZ1 and SpSIZ1 using MEGA 7 and genedoc software.


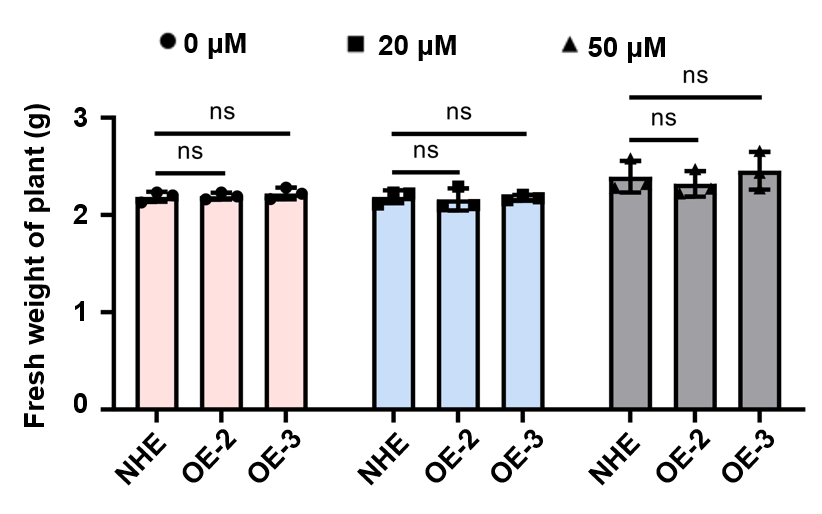


**Figure S2.** The fresh weight of NHE and SpSIZ1-OE lines before different concentrations of Cd stress treatments. Data are shown as mean ± SD (n = 3). ns indicate not statistically significant differences compared with wild-type NHE.


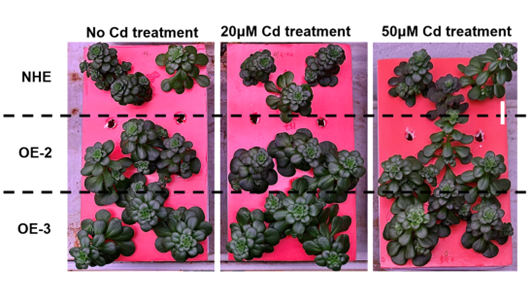


**Figure S3.** Treatment of different concentrations of Cd stress on the NHE and SpSIZ1-OE lines in the same cultivation environment. The CdCl_2_ stress conditions were 20 μM for 10 d and 50 μM for 10 d. A 10-d period without Cd treatment was used as a negative control. Scale bar, 5 cm.


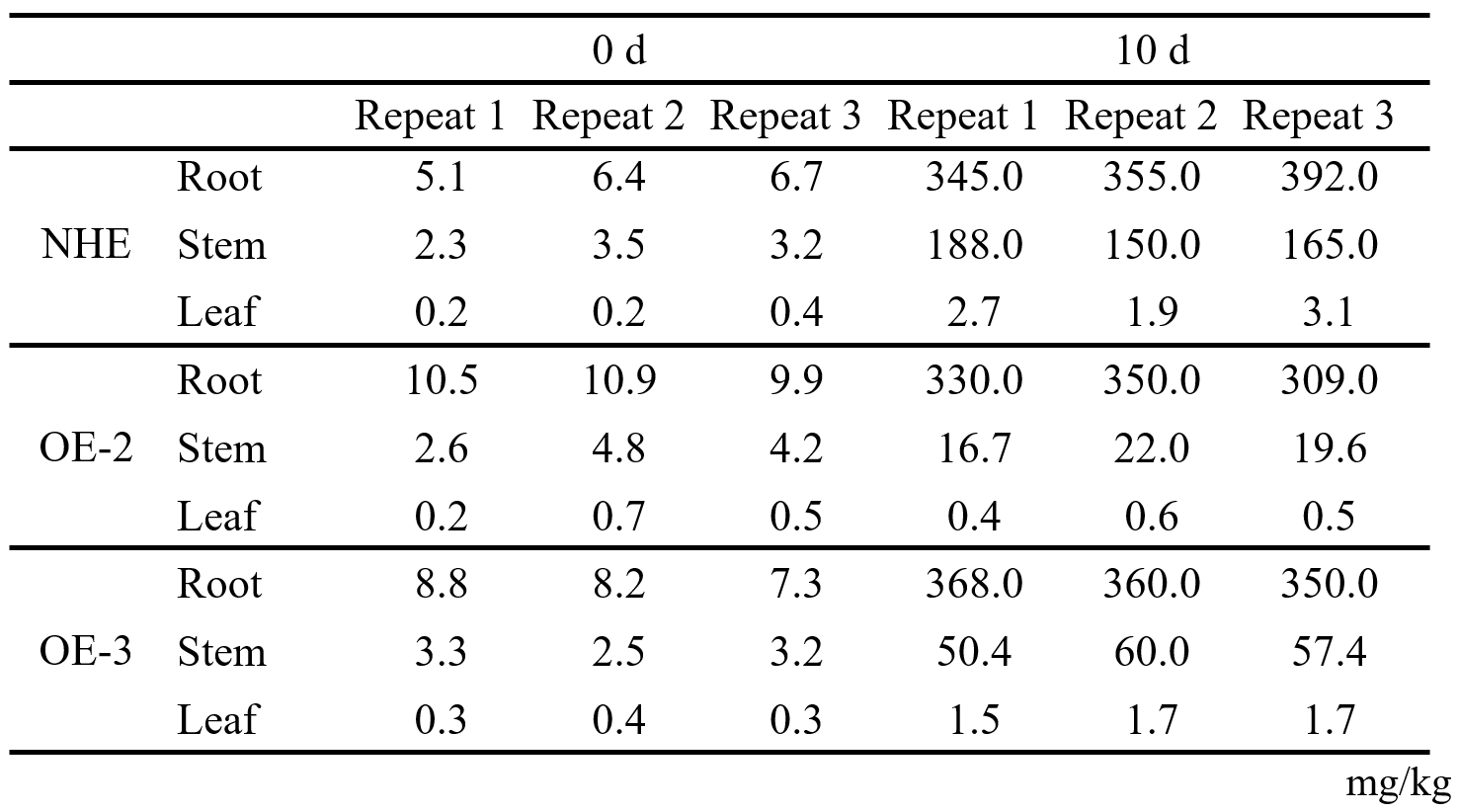


**Table S1.** The Cd content in the root, stem, and leaf of NHE and SpSIZ1-OE lines after 0 d and 10 d treatment with 50 μM CdCl_2_.


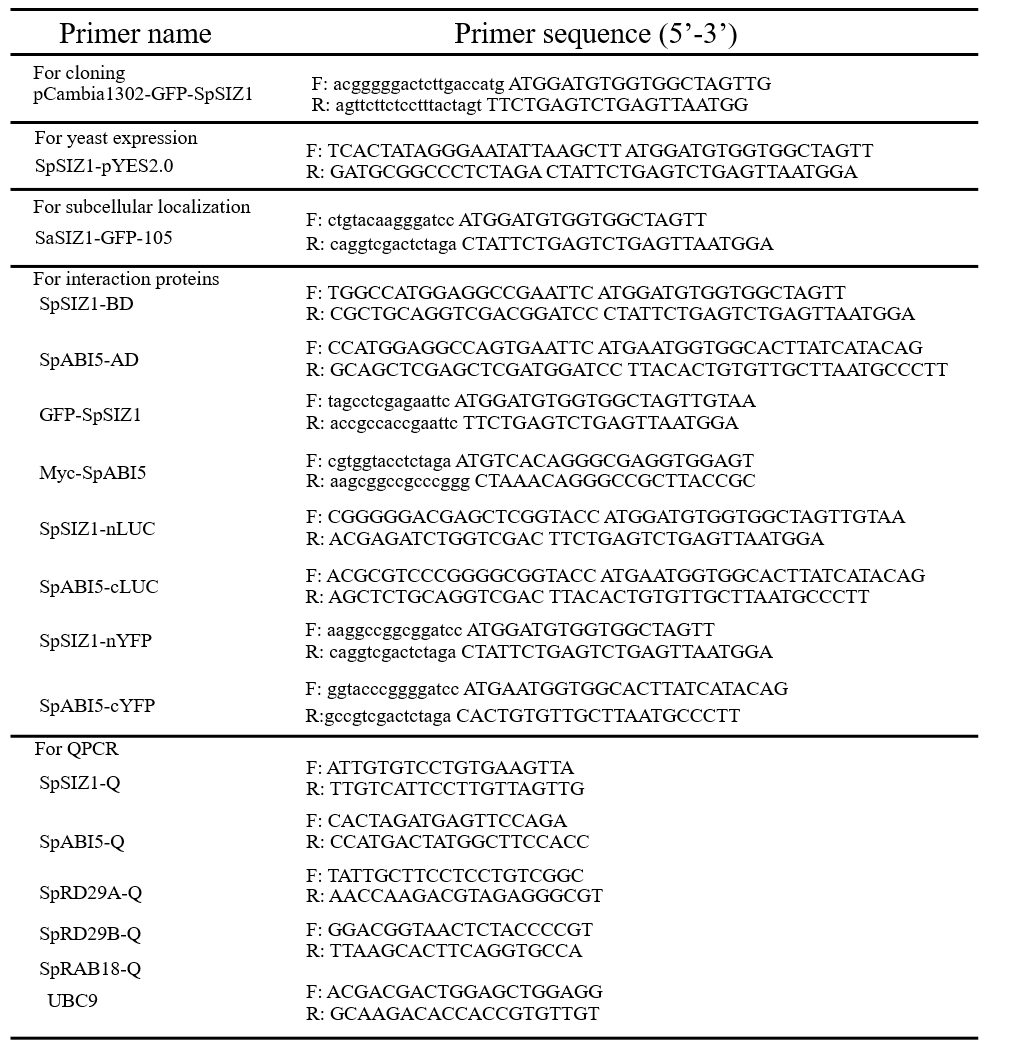


**Table S2.** Sequences of primers used in this study.
